# Phylogenetic relationships of typical antbirds (Thamnophilidae) and test of incongruence based on Bayes factors

**DOI:** 10.1186/1471-2148-4-23

**Published:** 2004-07-30

**Authors:** Martin Irestedt, Jon Fjeldså, Johan AA Nylander, Per GP Ericson

**Affiliations:** 1 Department of Vertebrate Zoology and Molecular Systematics Laboratory, Swedish Museum of Natural History, P.O. Box 50007, SE-104 05, Stockholm, Sweden; 2 Department of Zoology, University of Stockholm, SE-106 91 Stockholm, Sweden; 3 Vertebrate Department, Zoological Museum, University of Copenhagen, Universitetsparken 15, DK-2100 Copenhagen Ø, Denmark; 4 Department of Systematic Zoology, Evolutionary Biology, Centre, Uppsala University, Uppsala, Sweden

## Abstract

**Background:**

The typical antbirds (Thamnophilidae) form a monophyletic and diverse family of suboscine passerines that inhabit neotropical forests. However, the phylogenetic relationships within this assemblage are poorly understood. Herein, we present a hypothesis of the generic relationships of this group based on Bayesian inference analyses of two nuclear introns and the mitochondrial cytochrome *b *gene. The level of phylogenetic congruence between the individual genes has been investigated utilizing Bayes factors. We also explore how changes in the substitution models affected the observed incongruence between partitions of our data set.

**Results:**

The phylogenetic analysis supports both novel relationships, as well as traditional groupings. Among the more interesting novel relationship suggested is that the *Terenura *antwrens, the wing-banded antbird (*Myrmornis torquata*), the spot-winged antshrike (*Pygiptila stellaris*) and the russet antshrike (*Thamnistes anabatinus*) are sisters to all other typical antbirds. The remaining genera fall into two major clades. The first includes antshrikes, antvireos and the *Herpsilochmus *antwrens, while the second clade consists of most antwren genera, the *Myrmeciza *antbirds, the "professional" ant-following antbirds, and allied species. Our results also support previously suggested polyphyly of *Myrmotherula *antwrens and *Myrmeciza *antbirds. The tests of phylogenetic incongruence, using Bayes factors, clearly suggests that allowing the gene partitions to have separate topology parameters clearly increased the model likelihood. However, changing a component of the nucleotide substitution model had much higher impact on the model likelihood.

**Conclusions:**

The phylogenetic results are in broad agreement with traditional classification of the typical antbirds, but some relationships are unexpected based on external morphology. In these cases their true affinities may have been obscured by convergent evolution and morphological adaptations to new habitats or food sources, and genera like *Myrmeciza *antbirds and the *Myrmotherula *antwrens obviously need taxonomic revisions. Although, Bayes factors seem promising for evaluating the relative contribution of components to an evolutionary model, the results suggests that even if strong evidence for a model allowing separate topology parameters is found, this might not mean strong evidence for separate gene phylogenies, as long as vital components of the substitution model are still missing.

## Background

The typical antbirds (Thamnophilidae) is a speciose family within the furnariid radiation (sensu [1]) of the New World suboscine clade. The family includes fully 200 species [2] that all are restricted to neotropical forests. Most species are arboreal or undergrowth inhabitants, while only a few members are clearly terrestrially adapted, which otherwise seems to be the commonest lifestyle for most members in closely related clades (e.g., gnateaters Conopophagidae, antpittas Grallariidae, tapaculos Rhinocryptidae, and antthrushes Formicariidae). The highest diversity of typical antbirds is found in the Amazonian basin, and differences in ecological specializations make it possible to find as many as 40 species in the same area [3]. Morphologically typical antbirds shows considerable variation in size and patterns and colors of the plumage (black and shades of grey, buff and chestnut, with sexual plumage dimorphism in many species), while the variation in shape is more restricted. Many insectivorous niches are occupied, but the specialization of some species to follow army ants (to capture escaping insects) is perhaps the most well known. This habit has also given raise to the vernacular family name.

In traditional classifications, the antpittas (Grallariidae) and antthrushes (Formicariidae) were grouped together with typical antbirds in an even larger family. However, the support for the expanded antbird family was indeed weak, and both morphological [4-6] and molecular [1,7] evidence suggests that antpittas and antthrushes are distantly related to typical antbirds. DNA sequence data [1,8] suggests that gnateaters (Conopophagidae) forms the sister clade to typical antbirds, while antpittas and antthrushes are more closely related to tapaculos (Rhinocryptidae), woodcreepers and ovenbirds (Furnariidae).

Even though the monophyly of typical antbirds seems to be well supported by both syrinx morphology [6] and molecular data [1,7] the phylogenetic relationships within this assemblage are poorly understood, and the confusion extending to all taxonomic levels. Both the monophyly of several genera of typical antbirds has been questioned [3,9,10], as well as the delimitation of certain species [2,11-14]. Some species have also been moved from one genus to another (e.g., the black-hooded antwren that has been moved from the genus *Myrmotherula *to *Formicivora *[15]). The current knowledge of the phylogenetic relationships among typical antbirds rests mainly on interpretations drawn from external features, mostly of bill and feet, and has remained essentially the same for 150 years [2].

As typical antbirds are morphologically and ecologically diverse, they form a challenging group for studies of, e.g. adaptive evolution. However, such studies, as well as biogeographic interpretations, are difficult to make as long as there is no phylogenetic hypothesis. The aim of this study is therefore to create a hypothesis of generic relationships of typical antbirds that could be used as a framework for more detailed studies of the evolution of the group. Two nuclear introns, intron 2 in myoglobin and intron 11 in the glyceraldehyde-3-phosphodehydrogenase gene (G3PDH), and the mitochondrial cytochrome *b *gene, have been sequenced for 51 typical antbird taxa representing 38 out of the 45 genera recognized by Ridgely and Tudor [3]. We have used Bayesian inference and Markov chain Monte Carlo (MCMC) to estimate the phylogenetic relationships.

A common assumption made by molecular systematists is that gene trees accurately reflect species trees. Nevertheless, different data partitions may have different phylogenies due to processes as lineage sorting, gene duplication followed by extinction, and lateral transfer by hybridization and introgression (reviewed in [16-18]).

Primarily, there are two contradictory strategies utilized to handle data sets with significant phylogenetic incongruence between independent data partitions. Advocates for a "total evidence approach" (e.g., [19,20]) suggest that available data always should be combined, even though individual data partitions might be partly incongruent. The arguments are that a combination of different data partitions might improve the total resolution as different data partitions might be useful to resolve different areas of the tree, and that additive data sets might enhance phylogenetic informative characters that have been hidden by noise in the individual partitions. Opponents to this view (e.g., [21,22]) advice that data partitions with a significant level of incongruence should not be combined, as reliable characters might be obscured by random or systematic errors and in the worse case result in an erroneous topology (even though individual data partitions might provide consistent estimates).

However, when independent evidence is lacking and incongruence occurs between individual data partitions, it may be difficult to determine whether particular partitions are better estimates of the species tree than others. Researchers might favor the "total evidence approach" for this particular reason (even though the argument for not combining data partitions with significant levels of incongruence have strong merits). However, the degree of incongruence between individual gene trees could be used to determine whether the phylogenetic conclusions should be based on the combined data set, or only those parts that are similar among the different partitions. A commonly used approach for analysing combined data with maximum likelihood is to assume a single (the same) substitution model for all of the combined genes (for exceptions, see [23,24]). A significant result of incongruence between the combined result and the individual genes can then be hard to explain, since the incongruence could be due to both true difference in gene phylogeny and a misfit in the assumed model of evolution for the combined data [21,25]. This misfit could, for example, be a result of not allowing a heterogeneous model, that is, not allowing the different genes to have separate substitution models in the combined analysis [26]. We have thus explored our data partitions (the individual genes) by the congruence test described by Nylander et al. [27], which utilizes Bayes factors. The test is not an explicit significance test but compares the strength of evidence between two models of character evolution.

Although nuclear genes (as when situated on different chromosomes) may be considered as members of different linkage groups, the maternally inherited mitochondrial genome is effectively independent from the nuclear genome. Organelle genomes have also been suggested to more susceptible to "flow" between taxa during hybridization (although much less common in animals than in plants). In birds Degnan and Moritz [28] and Degnan [29], for example, have demonstrated that the mitochondrial tree in Australian white-eyes misrepresented the tree of nuclear loci and the expected species tree, possibly due to previous hybridization events. We have thus primarily been interested in the potential incongruence between the mitochondrial cytochrome *b *and the two nuclear genes (myoglobin and G3PDH), but all combinations of the three genes were examined. However, limitations in the substitution models might be the most important explanation to observed incongruence between data partitions, rather than an intrinsic phylogenetic incongruence [27]. We also explored how changes in substitution models affected the observed incongruence in our data set.

## Results

### Molecular variation and sequence distances

After alignment, the concatenated sequences become 2173 bp long. A total of between 679 bp (*Sclerurus scansor*) and 723 bp (*Myrmotherula leucophthalma*) was obtained from myoglobin intron 2, between 351 bp (*Rhegmatorina melanosticta*) and 400 bp (*Myrmeciza griseiceps*) from G3PDH intron 11, and 999 bp from cytochrome *b*. The observed, pairwise distances between ingroup taxa range between 0, 7% and 10, 7% in myoglobin, between 0, 3% and 19, 3% in G3PDH and between 6, 5% and 23, 9% in cytochrome *b*. Indels were found both in the myoglobin intron 2 and in the G3PDH intron 11. In most cases these are autapomorphic indels or occur in especially variable and repeatable regions. Given the tree topologies obtained from the Bayesian analyses, some synapomorphic indels were observed. For example, all *Thamnophilus *representatives share with *Sakesphorus bernardi *an insertion in the G3PDH intron, and, together with *Dysithamnus mentalis *and *Herpsilochmus atricapillus*, an insertion in the myoglobin intron.

### Phylogenetic inference and molecular models

**Figure 1:**
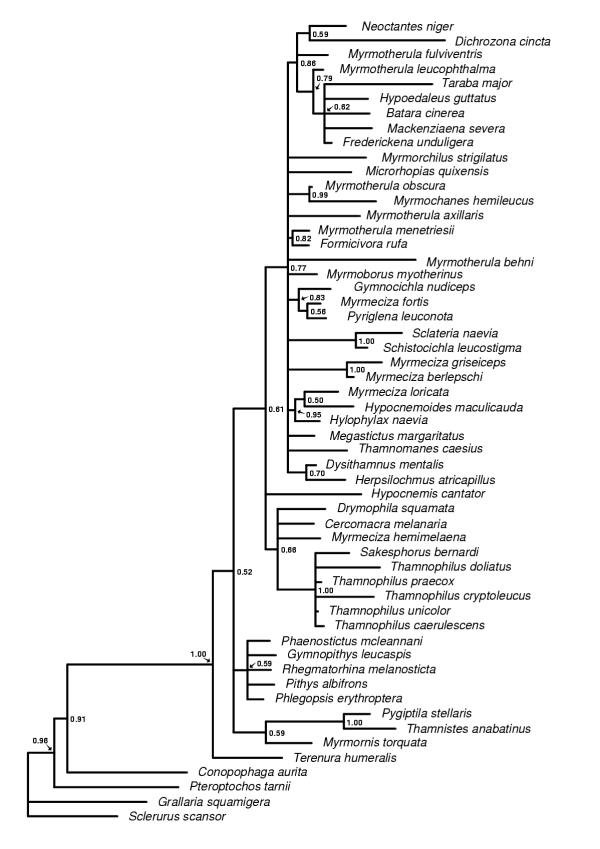
**The G3PDH majority rule consensus tree. **The 50% majority rule consensus tree obtained from the Bayesian analyses of the G3PDH (glyceraldehydes-3-phosphodehydrogenase) intron 11 data set. Posterior probability values are indicated to the right of the nodes.

**Figure 2:**
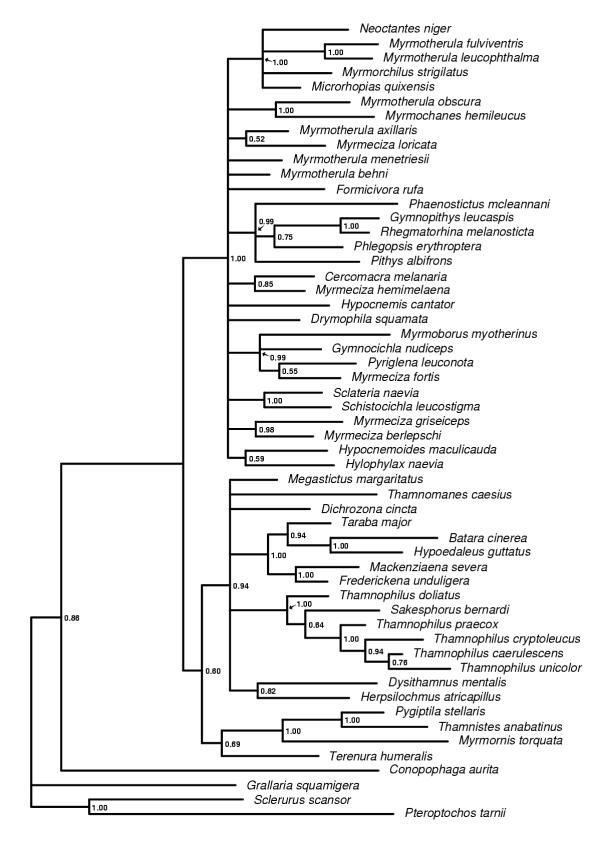
**The myoglobin majority rule consensus tree. **The 50% majority rule consensus tree obtained from the Bayesian analyses of the myoglobin intron 2 data set. Posterior probability values are indicated to the right of the nodes.

**Figure 3:**
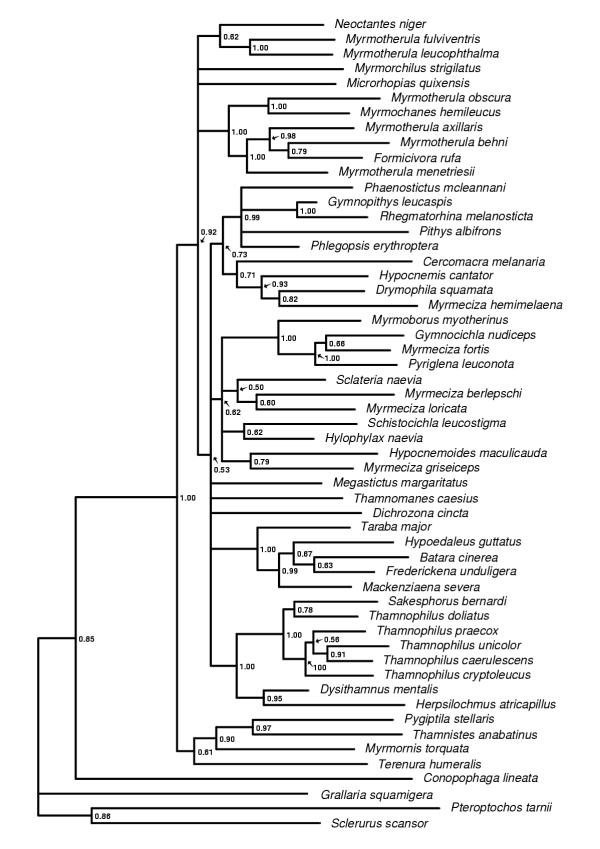
**The cytochrome b majority rule consensus tree. **The 50% majority rule consensus tree obtained from the Bayesian analyses of the cytochrome b data set. Posterior probability values are indicated to the right of the nodes.

**Figure 4:**
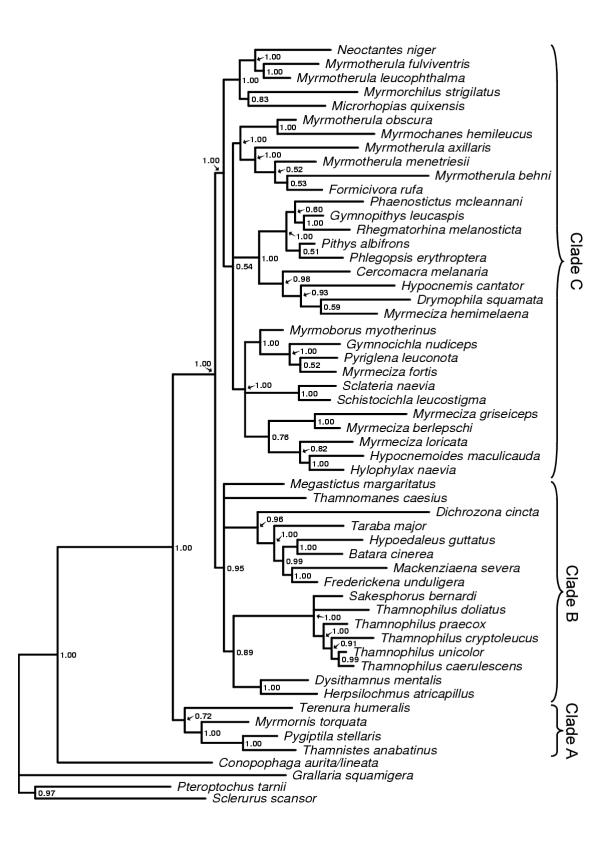
**The combined majority rule consensus tree. **The 50% majority rule consensus tree obtained from the analyses of the combined data set (G3PDH intron 11, the myoglobin intron 2 and the cytochrome b data sets). Clades A, B and C are major groups of typical antbirds discussed in the text. Posterior probability values are indicated to the right of the nodes.

A priori selection of substitution models showed that fairly parameter rich models were the best fit for all data partitions. Importantly, modeling rate variation seemed to be an important component. For the cytochrome *b *partition the GTR+I+Γ was the best fit, and for myoglobin intron 2, it was the GTR+ Γ. For the G3PDH intron 11 the somewhat simpler HKY+ Γ model was chosen. These models were used in the consecutive MCMC of the individual genes as well in the combined analysis. The parameter estimates from the two separate MCMC runs for each data set were found to be very similar (data not shown), thus allowing an inference from the concatenated output. After discarding the burn-in phase the inference for the cytochrome *b *was based on a total of 36, 000 samples from the posterior, for myoglobin the inference was based on 38, 000 samples, and for G3PDH and the combined data, inference were based on 38, 000, and 55, 600 samples, respectively. For the phylogenetic inference, the mode of the posterior distribution of topologies was presented as a majority-rule consensus tree from each analysis (Figures 1,2,3,4).

The trees obtained from the Bayesian analyses of the individual genes (cytochrome *b*, myoglobin and G3PDH) and the combined data set all differ in topology and degree of resolution. The G3PDH gene produced the poorest resolved tree (Figure 1) and also contains the smallest number of nodes with posterior probability values above 0.90. The myoglobin (Figure 2) and cytochrome *b *(Figure 3) genes produced trees with similar degree of resolution and nodal supports, but there is a weak tendency for cytochrome *b *giving better resolution and support at terminal nodes. The combined data set (cytochrome *b*, myoglobin and G3PDH) produced the most resolved tree (Figure 4) with the highest number of strongly supported nodes (exceeding 0.90 posterior probability). Overall, the myoglobin, the cytochrome *b *and the combined trees are topologically rather similar, while the G3PDH tree is the most deviant. A common pattern in all trees is that several nodes are unresolved, or short with low or intermediate posterior probabilities support values (0.50–0.90). The observed topological conflicts between the obtained trees generally occur at these short nodes, and there are only a few nodes with posterior probabilities values above 0.90 that are in conflict between the trees. Of these, one concerns the outgroup relationships (the G3PDH tree supports with 0.96 posterior probability a position of *Pteroptochos tarnii *that differs from all other trees). The other two conflicts concern internal relationships within well supported sub-clades: The cytochrome *b *tree places with 0.98 posterior probability *Myrmotherula menetriesii *basal to a clade consisting of *Myrmotherula axillaris, Myrmotherula behni *and *Formicivora rufa*. In the combined tree *Myrmotherula menetriesii *instead is nested within this clade with 1.00 posterior probability. The myoglobin tree suggests with 0.94 posterior probability that *Taraba major *is basal to *Batara cinerea *and *Hypoedaleus guttatus*, while *Taraba major *is basal also to *Mackenziaena severa *and *Frederickena unduligera *with 0.99 posterior probability in both the combined and the cytochrome *b *trees.

However, most suggested relationships are congruently supported by more than one of the trees obtained from the individual genes and by the combined data set. Several clades are also supported by all three genes trees as well as by the combined data set, including the recognition of a monophyletic origins of 1) the "large antshrikes" (*Taraba major*, *Batara cinerea*, *Hypoedaleus guttatus*, *Mackenziaena severa*, and *Frederickena unduligera*), 2) the "professional" ant-following antbirds (*Pithys albifrons, Phlegopsis erythroptera*, *Phaenostictus mcleannani*, *Rhegmatorhina melanosticta *and *Gymnopithys leucaspis*), 3) a *Sakesphorus*-*Thamnophilus *antshrike lineage (*Sakesphorus bernardi *and the five representatives of the genus *Thamnophilus*), and 4) a clade consisting of the wing-banded antbirds (*Myrmornis torquata*), the spot-winged antshrike (*Pygiptila stellaris*) and the russet antshrike (*Thamnistes anabatinus*). Sistergroup relationships between antvireos (*Dysithamnus mentalis*) and *Herpsilochmus *antwrens (*Herpsilochmus atricapillus*), as well as between *Myrmotherula obscura *and *Myrmochanes hemileucus *are also recognized by all trees.

Based on the tree obtained from the Bayesian analysis of the combined data set, typical antbirds could also be divided into three major clades (marked as A, B and C in Figure 4). The first clade (clade A) includes four genera that are suggested to have a basal position in relation to all other typical antbirds (1.00 posterior probability in the combined tree). This basal group (supported by 0.72 posterior probability in the combined tree) includes the representative of *Terenura *antwrens (*Terenura humeralis*), the wing-banded antbird (*Myrmornis torquata*), the spot-winged antshrike (*Pygiptila stellaris*) and the russet antshrike (*Thamnistes anabatinus*).

The second clade (clade B, Figure 4) is supported by 0.95 posterior probability in the combined tree and includes all antshrike genera (except the spot-winged antshrike and the russet antshrike, see clade A), antvireos (*Dysithamnus*), *Herpsilochmus *antwrens and the banded antbird (*Dichrozona cincta*). Within this large clade several lineages occur that receives more than 0.95 posterior probability. Noticeable within this clade is that neither the analyses of the individual genes nor the combined data set conclusively support that the representative of the antshrike genus *Sakesphorus *(*Sakesphorus bernardi*) is phylogenetically separated from the *Thamnophilus *antshrikes.

The last clade (clade C, Figure 4), including the *Myrmeciza *antbirds, most antwren genera (e.g., *Myrmotherula *and *Formicivora*), the "professional" ant-following antbirds, and some allied species, is supported by a 1.00 posterior probability value. Also within this clade several lineages are supported by posterior probability values above 0.90. However, the most interesting observation is the strong support for a polyphyletic origin of the *Myrmeciza *antbirds and the *Myrmotherula *antwrens.

### Tests of incongruence

The Bayes factor tests showed extensive incongruence between partitions, at least in the sense that relaxing the assumption of a common topology parameter always gave a better model likelihood (Table 1). For example, allowing the cytochrome *b *partition to have a separate topology from the two nuclear partitions myoglobin and G3PDH, gave a 2logB_12 _of 60.8. This value strongly suggests that an unlinked model is superior to the model assuming a common topology parameter for all partitions. This would also suggest that there is strong conflict between the mitochondrial and the nuclear partitions. However, this conclusion is far from conclusive when we consider the linking of the topology parameter for other combinations of the data. Combining the topology parameter for either one of the nuclear partitions with the mitochondrial, actually gives a better model (higher Bayes factors) than considering the mitochondrial vs. the nuclear partition (Table 1). For example, compared to the model that assumes a common topology parameter, unlinking the myoglobin partition from the other gave a 2logB of 102.26. Unlinking the G3PDH partition gave an even better model, with a 2logB of 118.12. Furthermore, if we would have to choose the one partitioning scheme that had the highest model likelihood, the model allowing a separate topology parameter for all partitions would be the clear choice (having a 2logB of 241.36 compared to the common model). 

**Table 1:** Summary of Bayes factor tests of incongruence. Entries are twice the log of the Bayes factor in the comparison between models M_1 _and M_2 _(2log*B*_12_). The row models are arbitrarily labeled M_1_; thus, positive values indicate support for the column model over the row model. A dash (-) indicates which partitions that have linked topology parameters.

Model	Cyt b-Myo-G3PDH	Cyt b, Myo-G3PDH	Cyt b-Myo, G3PDH	Cyt b-G3PDH, Myo	Cyt b, Myo, G3PDH
Cyt b-Myo-G3PDH	0	60.84	118.12	102.26	241.36
Cyt b, Myo-G3PDH		0	57.28	41.42	180.52
Cyt b-Myo, G3PDH			0	-15.86	123.24
Cyt b-G3PDH, Myo				0	139.1
Cyt b, Myo, G3PDH					0

The parsimony based ILD-test did not find a significant incongruence between the three gene partitions (p = 0.967).

## Discussion

### Phylogenetic incongruence between gene partitions

Allowing the gene partitions to have separate topology parameters clearly increased the model likelihood. That is, the unlinked models clearly had a better fit to the data than the linked models. Judging from the absolute value of the 2logB (Table 1), we are inclined to conclude that we should treat each partition as having its own posterior distribution of trees. However, the question is if we from these results really can say that the gene partitions evolved on different phylogenies? There are several reasons why different data partitions may have different phylogenies, although being sampled from the same taxa, or even the same individuals (se above). We cannot completely rule out the occurrence of any of these processes in our data. However, we believe that the interpretation based solely on Bayes factors might be hazardous. For instance, is it plausible that all three gene partitions had evolved on three different phylogenies, or that the linking of cytochrome *b *and myoglobin is a more reasonable partition of the data, instead of the mitochondrial versus the nuclear partitions? Nylander et al. [27], speculate that limitations in the substitution models might be more reasonable explanations to the high Bayes factors observed when comparing unlinked and linked models. Changing a component of the nucleotide substitution model, e.g. adding parameters to model rate variation, had much higher impact on the model likelihood than unlinking parameters among data partition. To illustrate the impact of changing the substitution model in our data, we run additional MCMC analyses under a different set of models, and compared them with the previous analyses using Bayes factors. The results were striking (Table 2). For example, we compared two models without rate variation, one with linked and the other with unlinked topologies (in both models GTR was used for cytochrome *b *and for myoglobin, and HKY for the G3PDH). The 2logB was 295.98 in favor for the unlinked model. However, adding parameters for modeling rate variation to either of the two models increased the model likelihood tremendously. The 2logB in favor of a model having parameters for rate variation (applying the same substitution models as the ones chosen a priori using AIC, see material and methods), varied between 5125.22 and 5662.56, depending on the model being compared (Table 2). Similar observations of magnitude changes in Bayes factors were made by Nylander et al. [27], when allowing rate variation. Another striking feature was that once parameters for modeling rate variation had been incorporated into the model, unlinking topologies did not seem to have as pronounced effect on the model likelihood (Table 2), compared to the models without rate variation. This observation is in concordance with previous findings that many functional genes have a strong among-site rate variation and that adding the relevant parameters to the model is likely to have a large effect on the likelihood [23,27,30,31].

**Table 2:** Summary of Bayes factor tests showing the effect of changing substitution model components. Entries are twice the log of the Bayes factor in the comparison between models M_1 _and M_2 _(2log*B*_12_). The row models are arbitrarily labeled M_1_; thus, positive values indicate support for the column model over the row model. A dash (-) indicates which partitions that have linked topology parameters. Asterisks (*) indicate models where the rates are assumed to be equal.

Model	Cyt b-Myo-G3PDH	Cyt b, Myo, G3PDH	Cyt b-Myo-G3PDH*	Cyt b, Myo, G3PDH*
Cyt b-Myo-G3PDH	0	241.36	-5421.2	-5125.22
Cyt b, Myo, G3PDH		0	-5662.56	-5366.58
Cyt b-Myo-G3PDH*			0	295.98
Cyt b, Myo, G3PDH*				0

It is worth noting that the parsimony based ILD-test did not find a significant incongruence between the three gene partitions. The value of this observation is uncertain, however, as the ILD test is based on another optimality criterion (parsimony). Furthermore, the strength of the test and interpretation of the results have also been questioned (e.g., [32])

In conclusion, allowing partitions to have separate topology parameters put fewer restrictions on the data. Hence, we should expect to find a better fit of the model to the data. Bayes factors seem promising for evaluating the relative contribution of components to an evolutionary model. However, judging from the relative increase in model likelihood when unlinking topologies compared to e.g., adding parameters for rate variation, we would anticipate components in the substitution model (for example, allowing rate variation among lineages) to have more effects on accommodating incongruence in the data. That is, even if we find strong evidence for a model allowing separate topology parameters, this might not mean strong evidence for separate gene phylogenies, as long as vital components of the substitution model are still missing. For further discussions on Bayesian approaches to combined data issues see e.g., [25,26,33].

### Phylogeny and morphological variation in typical antbirds

Even though we are unable to conclusively tell whether the observed phylogenetic incongruence between the individual gene partitions is due to genuine differences in phylogeny, or to limitations in the models used, we believe that the tree obtained from the combined data set represents the best estimate of the true relationships within the typical antbird assemblage. Obviously, several relationships are strongly supported, by congruent recognition by the individual gene trees and/or by high nodal support values. Nevertheless, other relationships have to be regarded as tentative, and especially those where any of the individual gene trees gives a strong nodal support for an alternative topology.

It is noticeable that, although the individual genes congruently support several terminal groups, basal relationships are generally less well resolved and more often in conflict. Even though this observation might be biased due to the use of improper molecular models when calculating the trees, biased mutation rate in studied genes, or a biased taxon sampling, it could indicate that the diversifications of typical antbirds was characterized by some rapid speciation bursts. There are only a few recent studies of typical antbirds with taxon samplings that includes representatives from several genera, but these studies show similar difficulties in resolving generic relationships. For example, in a study of phylogenetic relationships of *Myrmotherula *antwrens that included representatives from several other typical antbird genera, Hackett and Rosenberg [10] obtained considerably different topologies from plumage characters, allozyme and morphometric data, respectively. In addition, the phylogenetic relationships suggested from mitochondrial DNA sequence data within a partly comparable taxon sampling [9], have little resemblance to those in Hackett and Rosenberg [10]. The nodes between typical antbirds in the DNA-DNA hybridization "tapestry" by Sibley and Ahlquist [[7]: Figure 372] also contain a high degree of short branches.

It is also apparent that earlier antbird taxonomists, using external morphology, had difficulties in their taxonomic decisions and interpretations of higher-level relationships. Ridgway [[34]: p. 9] expressed that "The classification of this group is very difficult, more so probably than in the case of any American family of birds". Hackett and Rosenberg [10] concluded that antwren speciation mainly has been followed by plumage differentiation (and to some degree size differentiation) rather than changes in body proportions. Overall, this evolutionary pattern, with great changes in plumage and more limited changes in body proportions, seems to characterize the entire typical antbird assemblage (in contrast to the situation in ovenbirds, where there is a great variation in body proportions but not in plumage characters). However, Hackett and Rosenberg [10] suggested that neither plumage nor morphometric data correctly predicted the genetic relationships among the studied taxa. Our results seem to support their assumption as the traditionally used plumage characters in typical antbirds, as stripes, wingbars, and general coloration; seem to be irregularly distributed in the phylogenetic tree. It is reasonable to assume that plumage characters in typical antbirds are variable to such a degree that they are of limited use in studies of higher-level relationships. High levels of homoplasy (convergences and reversals) in plumage characters have also been reported in other passerine birds e.g., in Australian scrubwrens [35] brush-finches [36], and in New World orioles [37].

However, if excluding members in the "basal" group (clade A, Figure 4) and a few other aberrant taxa, the division of typical antbirds into the two main lineages in our phylogeny (clade B and C, Figure 4) is overall in good agreement with their body proportions (although there is a considerable size variation within both clades). The antshrikes (excluding *Tamnistes *and *Pygiptila*), antvireos and *Herpsilochmus *antwrens in clade B (Figure 4) are all more or less robust birds with heavy and prominently hooked bills, and many of them have a barred plumage pattern. The taxa in clade C (Figure 4), which includes most antwren genera, the *Myrmeciza *antbirds, the "professional" ant-following antbirds and some allied species, are generally slimmer birds with longer, thinner bills that have a less prominent hook. Most suggested relationships within clade B and C are in good agreement with traditional classifications. The recognition of monophyletic origins of most of the "professional" ant-following taxa (*Phaenostictus*, *Gymnopithys*, *Rhegmatorhina*, *Pithys *and *Phlegopsis*) and the "large" antshrikes (*Taraba*, *Hypoedaleus*, *Batara*, *Frederickena *and *Mackenziaena*) are two examples where our results are congruent with traditional classifications. The suggested relationships between the *Hypocnemis *and *Drymophila *antbirds, and the *Herpsilochmus *antwrens and the antvireos (*Dysithamnus*), respectively, have also been proposed previously based on molecular data [9,10]. Unfortunately, the genera *Biatas*, *Clytoctantes*, *Percnostola*, *Rhopornis*, *Stymphalornis *and *Xenornis *were lacking in our study; while most of these should probably be referred to Clade C, *Biatas *is difficult to place.

### Some novel relationships and the phylogenetic positions of some aberrant taxa

For certain taxa the position in our combined phylogeny is unexpected considering the external morphology and traditional classification. Most noticeable are the position of the banded antbird (*Dichrozona cincta*), which is nested within the clade with antshrikes, antvireos and *Herpsilochmus *antwrens (clade B, Figure 4), and the position of the wing-banded antbird (*Myrmornis torquata*) as sister to the russet antshrike (*Thamnistes anabatinus*) and the spot-winged antshrike (*Pygiptila stellaris*) (clade A, Figure 4). However, the increased number of molecular based phylogenies in recent years have led to discoveries of several examples, at different phylogenetic levels, were birds have been misclassified due to significant morphological differences from the taxa to which they are most closely related [38-40].

The phylogenetic position of the wing-banded antbird (*Myrmornis torquata*) has long been obscured and it was long placed with the typical army-ant followers (e.g., [2]). The wing-banded antbird has also been suspected to be related to ground antbirds (Formicariidae sensu [7]) based on similarities in morphology and general appearance [7]. Our results confidently place it within typical antbirds, a conclusion further supported by its vocalization [2] and choice of nest site and its white egg [41]. The well supported relationship to the arboreal russet antshrike (*Thamnistes anabatinus*) and spot-winged antshrike (*Pygiptila stellaris*), suggested by our data, has apparently been obscured by structural differences caused by its adaptation to a terrestrial life-style shared with for example the antthrushes. A similar explanation may apply to the peculiar position of the banded antbird (*Dichrozona cincta*) in the combined phylogeny, as this taxon is also a mainly terrestrial bird, unlike the other members in the "antshrike" clade (clade B, Figure 4). The fact that the banded antbird has a rather long branch in the combined tree and that its phylogenetic position alter between the individual gene trees, leads us to consider the phylogenetic position of the banded antbird (*Dichrozona cincta*) as preliminary. However, it is obvious that it is not closely related to the *Hylophylax *antbirds with which it has traditionally been grouped (based on similarities in plumage patterns and weak sexual dimorphism). It should be noted that, due to the peculiar position of *Dichrozona cincta*, a second individual (ZMUC 128217) have been sequenced for all three genes. There were no variation at all found between the two individuals in G3PDH, in myoglobin 1 ambiguous position were found, and in cytochrome *b *24 base pairs (2.4%) that differed as well as 3 ambiguous positions were found. Overall, this variation is within the variation that could be suspected between individuals within a species. Thus, the strange position of *Dichrozona cincta *in our analyses is unlikely to be due to sample or sequence mix-up.

There are several other, less striking examples where the position of taxa in our phylogeny conflicts with relationships suggested in classifications based on external morphology. The *Herpsilochmus *antwrens for example (traditionally placed among *Myrmotherula*, *Microrhopias *and *Formicivora *antwrens), are quite different in appearance from their sister group *Dysithamnus *in being rather slim, lacking a particularly hook-bill, and in having a distinctly patterned plumage (however, as discussed above a close relationship between *Herpsilochmus *and *Dysithamnus *is also supported by an independent molecular study). Other examples are the positions of *Myrmorchilus *and *Neoctantes*, respectively (see discussion below). In these cases their true affinities may have been obscured by morphological adaptations to habitats or food sources that differ from those preferred by their closest relatives.

The strong support in the combined tree for basal positions of *Myrmornis*, *Pygiptila, Thamnistes *and *Terenura *relative to all other typical antbirds is maybe the most unexpected result of our study. In a majority of classifications *Terenura *is placed close to other antwrens, but with no strong data support. Although the precise position of the *Terenura *antwrens is partly ambiguous in our analysis, they obviously belong to an ancient radiation that is only distantly related to the other "antwrens". The *Terenura *antwrens differ from other "antwrens" in plumage pattern and in being more slender and warbler-like with a thinner bill and longer tail. In a study based on mitochondrial DNA the position of *Terenura *was ambiguous depending on how the data set was analyzed [9] but clearly it was not closely related to the other taxa included in that study (e.g., *Myrmotherula*, *Formicivora, Herpsilochmus*, *Hypocnemis*, *Drymophila*).

The well-supported phylogenetic position of *Pygiptila *and *Thamnistes *as the sistergroup to *Myrmornis *(instead of being close to other antshrikes as suggested in many linear classifications), is novel. However, *Pygiptila *and *Thamnistes *resemble each other in their ways of feeding in the sub-canopy, *Thamnistes *also resembling the *Pygiptila *female in appearance, and differing from most antshrikes in feeding behavior. DNA-DNA hybridization data [7] and protein electrophoresis [10] have previously shown *Pygiptila *to be genetically distant from the *Thamnophilus *antshrikes. The general external resemblance of *Pygiptila *and *Thamnistes *to other antshrikes is therefore best explained, as being plesiomorphic, and this may also be the case with their suspended nest-type.

### *The polyphyly of *Myrmotherula *antwrens and *Myrmeciza *antbirds*

Our results confirm both previous molecular studies that suggest the *Myrmotherula *antwrens are polyphyletic [9,10], and the suspicion based on morphology that also the rather diverse genus *Myrmeciza *constitutes an unnatural taxon [3]. Nevetheless, most *Myrmeciza *antbirds studied herein belong to the same clade, although they are not monophyletic as several other genera (*Myrmoborus*, *Gymnocichla*, *Pyriglena*, *Sclateria*, *Schistocichla*, *Hypocnemoides*, and *Hylophylax*) are nested among them. However, the chestnut-tailed antbird (*Myrmeciza hemimelaena*), which represents a group of small and slim *Myrmeciza *antbirds with prominent wing spots in both sexes, groups with the *Drymophila*, *Hypocnemis *and *Cercomacra *antbirds. The small and slim *Myrmeciza *antbirds resembles morphologically the *Hypocnemis *antbirds in having similar wing spots as well as a rather short and rufous-brown tail.

The clade that includes the remaining *Myrmeciza *antbirds consists of three unresolved lineage. The first includes a group of large and heavily built *Myrmeciza *antbirds (represented by *Myrmeciza fortis*). Next outside this group is the fire-eye (*Pyriglena leuconota*), followed by the bare-crowned antbird (*Gymnocichla nudiceps*) and the *Myrmoborus *antbird representative (*Myrmoborus myotherinus*). These taxa have rather stout bodies and in most cases red eyes. Both the fire-eyes and the bare-crowned antbird were previously assumed to be related to the large, heavy-billed *Myrmeciza *antbirds (e.g., [3]). The second lineage consists of the silvered antbird (*Sclateria naevia*) and the *Schistocichla *antbird representative (*Schistocichla leucostigma*). These relationships are in good agreement with the overall plumage characters in these taxa [3], with the males being rather uniform gray while the females are rufous. Such a plumage is also found in the genus *Percnostola*, with which the *Schistocichla *antbirds are considered to be most closely related (*Schistocichla *and *Percnostola *have even been regarded as congeneric, but it has also been suggested that *Percnostola *could be polyphyletic).

In the third lineage, *Myrmeciza griseiceps *and *Myrmeciza berlepschi *form the sister clade to *Myrmeciza loricata, Hypocnemoides maculicauda *and *Hylophylax naevia *(the latter two are sister taxa). This group consists of rather typical shaped and sized "*Myrmeciza*" antbirds. Although it has a shorter tail, *Hylophylax naevia *shares plumage pattern with *Myrmeciza loricata *(*Hypocnemoides maculicauda *is more discretely patterned), while *Myrmeciza griseiceps *and *Myrmeciza berleschi*, on the other hand, are more uniformly colored birds.

A non-monophyletic origin of *Myrmotherula *antwrens, suggested by our data, agrees with the results of previous molecular studies [9,10]. The results also support Hackett and Rosenberg's [10] protein electrophoresis data suggesting that the "gray" and "streaked" forms of *Myrmotherula *antwrens are more closely related to each other than either is to the "checker-throated" forms. The combined tree (Figure 4, clade C) suggests that the *Myrmotherula *antwrens evolved along two separate phylogenetic lineages. In the first, the "checker-throated" forms (*Myrmotherula fulviventris *and *Myrmotherula leucophthalma*) group with the black bushbird (*Neoctantes niger*) and constitute the sister to the dot-winged antwren (*Microrhopias quixensis*) and the stripe-backed antbird (*Myrmorchilus strigilatus*). Based on the external morphology these taxa indeed constitute a rather heterogeneous group. For example, the stripe-backed antbird has previously been suggested to be related to *Formicivora *and *Drymophila *antwrens [42], which are distantly related according to our results. However, *Neoctantes*, *Microrhopias *and *Myrmorchilus *are monotypic genera that lack obvious close relatives. *Myrmorchilu*s is essentially a terrestrial bird, living in chaco scrub, thus differing in habits and habitat from the "typical" antwren lifestyle. *Neoctantes *lives in humid forest like most *Myrmotherula *antwrens, but its bill is modified to hammers on stems, vines etc., and to be used as a wedge to pry off strips of bark [2]. The morphological differences between *Neoctantes *and *Myrmorchilus *on one hand, and the "checker-throated" *Myrmotherula *antwrens on the other, could thus be the result of adaptive specializations in the former taxa.

In the second lineage of *Myrmotherula *antwrens, the "streaked" forms represented by the short-billed antwren (*Myrmotherula obscura*) and the black-and-white antbird (*Myrmochanes hemileucus*) form the sister group to the "gray" forms (represented by *Myrmotherula menetriesii*, *axillaris *and *behni*) and *Formicivora rufa*. Although the support for nesting *Formicivora rufa *among the "gray" forms of *Myrmotherula *is rather weak, it suggests that the generic boundary between *Formicivora *and "gray" *Myrmotherula *antwren is far from unambiguously settled. This is also indicated by the recent transfer of the black-hooded antwren from the genus *Myrmotherula *to *Formicivora *[15]. Bates et al. [9] also found a close relationship between *Myrmotherula longipennis *(belonging to the "gray" form of *Myrmotherula *antwrens) and the genus *Formicivora *(*Formicivora grisea *and *Formicivora rufa*).

## Conclusions

The phylogenetic results support that most antbirds could be divided into two major clades that are in broad agreement with traditional classifications. The first clade includes most antshrike genera, antvireos and the *Herpsilochmus *antwrens, while the second clade consists of the *Myrmeciza *antbirds, the "professional" ant-following antbirds, and allied. However, some relationships within these clades, as well as the support for that *Terenura *antwrens, the wing-banded antbird (*Myrmornis torquata*), the spot-winged antshrike (*Pygiptila stellaris*) and the russet antshrike (*Thamnistes anabatinus*) are basal to all other typical antbirds, are unexpected based on external morphology. Possibly the true affinities of these taxa have been obscured by morphological convergence due to adaptations to new habitats or food sources. Our results also strongly support that both the *Myrmeciza *antbirds and the *Myrmotherula *antwrens are unnatural groupings in need for taxonomic revisions. Also certain other taxa may be unnatural units, but definitive conclusions must await future analyses involving more taxa. 

Bayes factors seem promising for evaluating the relative contribution of components to an evolutionary model. However, changing a component of the nucleotide substitution model, e.g. adding parameters to model rate variation, had much higher impact on the model likelihood than unlinking parameters among data partition. Thus, even though strong evidence for a model allowing separate topology parameters is found, this might not mean strong evidence for separate gene phylogenies, as long as vital components of the substitution model are still missing.

## Methods

### Taxon sampling, amplification and sequencing

Totally 51 typical antbird species were selected for the molecular analyses, including representatives from 38 genera out of 45 genera recognized by Ridgely and Tudor [3]. From some antbird genera (*Myrmeciza*, *Myrmotherula *and *Thamnophilus*) several species were included, as the monophyly for these genera had been questioned [3,9,10]. The phylogenetic trees were rooted using representatives from major furnariid lineages suggested by Irestedt et al. [1]. Sample identifications and GenBank accession numbers are given in Table 3 (see additional file 1 ).

Nucleotide sequence data were obtained from two nuclear introns, myoglobin intron 2 and the glyceraldehydes-3-phosphodehydrogenase (G3PDH) intron 11, and from the mitochondrial cytochrome *b *gene. The complete myoglobin intron 2 (along with 13 bp and 10 bp of the flanking regions of exons 2 and 3, respectively) corresponding to the region between positions 303 (exon 2) and 400 (exon 3) in humans (GenBank accession number XM009949) and the complete G3PDH intron 11 (including 36 bp and 18 bp of exons 11 and 12, respectively) corresponding to the region 3915 to 4327 in *Gallus gallus *(GenBank accession number M11213) were sequenced. From the cytochrome *b *gene 999 bp were obtained corresponding to positions 15037 to 16035 in the chicken mitochondrial genome sequence [43]. Some indels were observed in the alignments of myoglobin intron 2 and the G3PDH intron 11, respectively (see results), but all gaps in the sequences were treated as missing data in the analyses. No insertions, deletions, stop or nonsense codons were observed in any of the cytochrome *b *sequences.

Extraction, amplification and sequencing procedures for cytochrome *b *and myoglobin intron 2 follow the descriptions in Ericson et al. [44] and Irestedt et al. [1]. A protocol described by Fjeldså et al. [45] was followed for the amplification and sequencing of the G3PDH intron.

For each gene and taxon, multiple sequence fragments were obtained by sequencing with different primers. These sequences were assembled to complete sequences with SeqMan II™ (DNASTAR inc.). Positions where the nucleotide could not be determined with certainty were coded with the appropriate IUPAC code. Due to a rather low number of insertions in myoglobin intron 2 and G3PDH intron 11 the combined sequences could easily be aligned by eye.

### Phylogenetic inference and model selection

We used Bayesian inference and Markov chain Monte Carlo (MCMC) for estimating phylogenetic hypothesis from DNA data (see recent reviews by Holder and Lewis, [46]; Huelsenbeck et al., [47]). Bayesian inference of phylogeny aims at estimating the posterior probabilities of trees and other parameters of an evolutionary model. Importantly, two components need to be specified (apart from the data): the model of nucleotide substitution and the prior distributions for the parameters in that model. The models for nucleotide substitutions were selected for each gene individually, prior to the MCMC, and using the Akaike Information Criterion (AIC [48]). This was done using the program MrModeltest [49] in conjunction with PAUP* [50]. Specifically, MrModeltest compares 24 standard substitution models, including models allowing rate variation, utilizing the likelihood scores calculated by PAUP* on an initial, approximate phylogeny (see e.g., [51]).

After models had been selected for the individual gene partitions, prior distributions for the model parameters were specified. For stationary state frequencies, we used a flat Dirichlet prior, Dir(1, 1, 1, 1). A Dirichlet prior, Dir(1, 1, 1, 1, 1, 1) were also used for the nucleotide substitution rate ratios of the general time-reversible model (GTR [52-54]). A Beta distribution, Beta(1, 1), were used for the transition/transversion rate ratio of the Hasegawa-Kishino-Yano model (HKY [55]). A uniform prior, Uni(0.1, 50), was used on the shape parameter of the gamma distribution of rate variation (Γ [56]), and a Uni(0, 1) prior was used for the proportion of invariable sites (I [57]). An exponential prior, Exp(10), were used for branch lengths, and all trees were assumed to be equally likely (flat prior on topology).

The posterior probabilities of trees and parameters in the substitution models were approximated with MCMC and Metropolis coupling using the program MrBayes [58]. The gene partitions were analyzed both separately and combined. In the combined analysis, each gene partition was allowed to have separate parameters by using a rate multiplier [27,58,59]. One cold and three incrementally heated chains were run for 3 million generations, with a random starting tree and a temperature parameter value of 0.2. Trees were sampled every 100th generations, and the trees sampled during the burn-in phase (i.e., before the chain had reached its apparent target distribution) were discarded. Two runs, starting from different, randomly chosen trees, were made to ensure that the individual runs had converged on the same target distribution [60]. Convergence of parameters was checked by examining parameter means and variances between runs. After checking for convergence, final inference was made from the concatenated output from the two runs.

### A Bayesian test of incongruence

Bayesian methods provide us ways not only to estimate posterior probabilities for trees and parameters in a model, but also to evaluate the model itself. Bayes factors [61], allow us to make sophisticated comparisons between models used in phylogenetic analyses [27,62,63]. Bayes factors measure the strength of evidence in favor of one model M_1 _compared to another M_2_, given the data X, and is calculated as the ratio of the model likelihoods, B_12 _= *f*(X|M_1_)/ *f*(X|M_2_). The model likelihoods *f*(X|M_*i*_) are difficult to calculate analytically but can be estimated by using the output from an MCMC [27,62].

Here we explore the congruence test described by Nylander et al. [27], which utilizes Bayes factors. The test is not a significance test but merely compares the strength of evidence between two models of character evolution. In the first model, data partitions are allowed to have their own unique set of substitution parameters, but we assume the data as having evolved on the same topology, but with partition-specific branch lengths. Strictly speaking, we are restricting the data partitions to have the same posterior distribution for topologies, but (potentially) different distributions in all other parameters. In the second model we relax the assumption of a single distribution of topologies for all data partitions. That is, if the data partitions (genes) truly evolved on different phylogenies, they are allowed to do so in the model. The comparison or 'test' is to see if the second model provides compelling evidence as to be accepted as superior. Here we use the log of the Bayes factor and a value of >10 for 2 logB_12 _have been suggested as strong evidence against the alternative model, M_2 _[61].

To accomplish the incongruence test we utilized the *unlink *command in MrBayes, which allows the user to let parameters as well as topologies to be unlinked between partitions. We calculated Bayes factors and compared the effects on the model likelihood when linking or unlinking topologies between all the gene partitions. We were primarily interested in the potential incongruence between the mitochondrial cytochrome *b *partition and the two nuclear partitions myoglobin and G3PDH, but all combinations of the three genes in our data set were examined.

For comparison, we also tested whether the different gene partitions were in significant conflict with each other by using the parsimony based incongruence-length differences test (ILD) [64], implemented in PAUP* [50]. The results are based on 10,000 replicates, with ten iterations (random additions of taxa) per replicate.

## Authors' contribution

MI designed the study, carried out the labwork, participated in the phylogenetic analyses, and drafted the manuscript. JF assisted with the design of the study and with the draft of the manuscript. JN performed the phylogenetic analyses, drafted parts of the results, and material and methods section of the manuscript. PE conceived the study. All authors read and approved the manuscript.

## Supplementary Material

Additional File 1 **Table 3. Samples used in the study**. The classification follows Ridgely and Tudor [3] for typical antbirds, and Irestedt et al. [1] for families. Abbreviations: AHMN = American Museum of Natural History, New York; FMNH = Field Museum of Natural History, Chicago; LSUMZ = Louisiana State University, Museum of Natural Science; NRM = Swedish Museum of Natural History; ZMCU = Zoological Museum of the University of Copenhagen. References: (1) Irestedt et al. [1]; (2) Fjeldså et al. [45]; (3) Johansson et al. [65]; Fjeldså et al. [66]. Click here for file
