# Supplementary material for: Phylogenetic relationships of typical antbirds (Thamnophilidae) and test of incongruence based on Bayes factors
Source: BMC Evol Biol. 2004 Jul 30;4:23. doi: 10.1186/1471-2148-4-23 (PMC509417; doi:10.1186/1471-2148-4-23)
Supplement: Additional File 1 — Table 3. Samples used in the study. The classification follows Ridgely and Tudor [3] for typical antbirds, and Irestedt et al. [1] for families. Abbreviations: AHMN = American Museum of Natural History, New York; FMNH = Field Museum of Natural History, Chicago; LSUMZ = Louisiana State University, Museum of Natural Science; NRM = Swedish Museum of Natural History; ZMCU = Zoological Museum of the University of Copenhagen. References: (1) Irestedt et al. [1]; (2) Fjeldså et al. [45]; (3) Johansson et al. [65]; Fjeldså et al. [66]. [file 1471-2148-4-23-S1.pdf]

Table 3. Samples used in the study. The classification follows Ridgely and Tudor [3] for typical antbirds, and Irestedt et al. [1] for families. Abbreviations: AHMN = American Museum of Natural History, New York; FMNH = Field Museum of Natural History, Chicago; LSUMZ = Louisiana State University, Museum of Natural Science; NRM = Swedish Museum of Natural History; ZMCU = Zoological Museum of the University of Copenhagen. References: (1) Irestedt et al. [1]; (2) Fjeldså et al. [45]; (3) Johansson et al. [65]; Fjeldså et al. [66].

Species	Sample no.	Cytochrome *b*	Myoglobin	G3PDH
Family Thamnophilidae				
Frederickena unduligera	ZMCU S1776	AY676934	AY676981	AY677028
Mackenziaena severa	NRM 956630	AY676935	AY676982	AY677029
Hypoedaleus guttatus	LSUMZ B-25895	AY676936	AY676983	AY677030
Batara cinerea	LSUMZ B-18607	AY676937	AY676984	AY677031
Taraba major	NRM 956694	AY676938	AY676985	AY677032
Sakesphorus bernardi	ZMCU S101	AY676939	AY676986	AY677033
Thamnophilus doliatus	NRM 956691	AY676940	AY676987	AY677034
Thamnophilus cryptoleucus	ZMCU S1196	AY676941	AY676988	AY677035
Thamnophilus praecox	ZMCU S108	AY676942	AY676989	AY677036
Thamnophilus unicolor	ZMCU S111	AY676943	AY676990	AY677037
Thamnophilus caerulescens	NRM 967007	AY078176 (ref. 2)	AY065783 (ref. 1)	AY336587 (ref. 3)
Megastictus margaritatus	ZMCU S2130	AY676944	AY676991	AY677038
Pygiptila stellaris	LSUMZ B-9703	AY676945	AY676992	AY677039
Thamnistes anabatinus	ZMCU S1607	AY676946	AY676993	AY677040
Thamnomanes caesius	ZMCU S1312	AY676947	AY676994	AY677041
Dysithamnus mentalis	NRM 956629	AY676948	AY676995	AY677042
Herpsilochmus atricapillus	ZMCU S1086	AY676949	AY676996	AY677043
Microrhopias quixensis	FMNH 321993	AY676950	AY676997	AY677044
Myrmotherula obscura	ZMCU S1836	AY676951	AY676998	AY677045
Myrmotherula leucophthalma	ZMCU S1306	AY676952	AY676999	AY677046
Myrmotherula fulviventris	ZMCU S1649	AY676953	AY677000	AY677047
Myrmotherula axillaris	ZMCU 126870	AY676954	AY677001	AY677048
Myrmotherula menetriesii	ZMCU 126444	AY676955	AY677002	AY677049
Myrmotherula behni	ZMCU 128008	AY676956	AY677003	AY677050
Terenura humeralis	FMNH 389941	AY676957	AY677004	AY677051
Drymophila squamata	ZMCU S2199	AY065722 (ref. 1)	AY065780 (ref. 1)	AY677052
Formicivora rufa	NRM 947236	AY676958	AY677005	AY677053
Myrmorchilus strigilatus	NRM 956742	AY676959	AY677006	AY677054
Cercomacra melanaria	NRM 947099	AY065723 (ref. 1)	AY065781 (ref. 1)	AY677055
Pyriglena leuconota	ZMCU S2007	AY065724 (ref. 1)	AY065782 (ref. 1)	AY677056
Neoctantes niger	FMNH 321806	AY676960	AY677007	AY677057
Myrmoborus myotherinus	ZMCU S1303	AY676961	AY677008	AY677058
Dichrozona cincta	ZMCU S1882	AY676962	AY677009	AY677059
Hylophylax naevia	ZMCU S1913	AY676963	AY677010	AY677060
Hypocnemis cantator	ZMCU S1300	AY676964	AY677011	AY677061
Myrmochanes hemileucus	LSUMZ B-7245	AY676965	AY677012	AY677062
Hypocnemoides maculicauda	ZMCU S1301	AY676966	AY677013	AY677063
Sclateria naevia	ZMCU S102	AY676967	AY677014	AY677064
Schistocichla leucostigma	ZMCU S1207	AY676968	AY677015	AY677065
Myrmeciza griseiceps	ZMCU S1221	AY676969	AY677016	AY677066
Myrmeciza hemimelaena	ZMCU 126372	AY676970	AY677017	AY677067
Myrmeciza loricata	ZMCU S1062	AY676971	AY677018	AY677068
Myrmeciza fortis	ZMCU 126346	AY676972	AY677019	AY677069
Myrmeciza berlepschi	ZMCU 126182	AY676973	AY677020	AY677070
Gymnocichla nudiceps	ZMCU S2732	AY676974	AY677021	AY677071
Myrmornis torquata	LSUMZ B-3228	AY676975	AY677022	AY677072
Pithys albifrons	ZMCU S1857	AY676976	AY677023	AY677073
Gymnopithys leucaspis	ZMCU S1843	AY676977	AY677024	AY677074
Rhegmatorhina melanosticta	ZMCU S1825	AY676978	AY677025	AY677075
Phlegopsis erythroptera	ZMCU S1860	AY676979	AY677026	AY677076
Phaenostictus mcleannani	ZMCU S1647	AY676980	AY677027	AY677077
Family Conopophagidae				
Conopophaga aurita	ZMCU S1245		AY065784 (ref. 1)	AY336577 (ref. 3)
Conopophaga lineata	NRM 956653	AY078173 (ref. 2)		
Family Rhinocryptidae				
Pteroptochos tarnii	AMNH RTC467	AY065717 (ref. 1)	AY065774 (ref. 1)	AY590096 (ref. 4)
Family Furnariidae				
Sclerurus scansor	NRM 937258	AY065715 (ref. 1)	AY065772 (ref. 1)	AY590080 (ref. 4)
Family Grallariidae				
Grallaria squamigera	ZMCU S78	AY065720 (ref. 1)	AY065778 (ref. 1)	AY677078
